# Nature and Treatment of Western Autumnal Diseases

**Published:** 1855-05

**Authors:** S. G. Armor

**Affiliations:** Prof. Pathology and Practice of Medicine in the Medical College of Ohio


### DEPARTMENT OF SELECTIONS.

### NATURE AND TREATMENT OF WESTERN AUTUMNAL FEVERS.

BY S. G. ARMOR, M. D.,

Prof. Pathology and Practice of Medicine in the Medical College of Ohio.

Fevers of a periodical type are peculiarly interesting to the Western and Southern practitioner, from the fact that they prevail in all parts of the United States lying between the Northern Lakes and the Gulf of Mexico. And although, in the main, there is a general agreement as to the principles of treatment, yet in the details of that treatment questions arise upon which there are widely different views. This results, doubtless, from different opinions as to the nature and pathology of these fevers; and to this question, therefore, I shall very briefly direct attention.

I have selected the Autumnal fevers that prevail so largely in the Mississippi Valley, as typical of the entire class of Essential fevers; differing, it is true, in many particulars from those that depend upon a specific animal poison, and yet in their general pathology offering good illustrations of the general class of Idiopathic fevers.

What, then, is the nature and pathology of these fevers? Shall we regard then as *constitutional* or as *local* maladies? What relation does the febrile or inflammatory action sustain to the local disease? Are the local alterations secondary to the fever, or are they symptomatic of the fever? Or is the fever symptomatic of them, as taught by Broussais? And what influence do they possess in inducing a fatal termination?

I briefly present here the prominent questions at issue; they are questions upon which volumes have been written; and unfortunately *theory* has too often taken the place of *fact*, until speculation itself has become weary of the investigation.

The theories of medicine have always and always will influence its practice. Thus the speculations of Brown, Cullen, Clutterbuck, Broussais, Rosari and Armstrong successively introduced the stimulant, diaphoretic, general antiphlogistic, leeching, tartar emetic and mercurial plans, each of which has, in its turn, been pushed to a most deleterious extent. The same may be affirmed of the theory of Dr. Cook, an influential teacher of the West, who appeared to see but one organ, the *Liver*, whose remedy, *Calomel*, has been used by his followers to a prodigal, and I may add, empirical and often cruel extent. It is important, therefore, in a practical point of view, that we should not reverse the Baconian method of arriving at the truth: our faith and practice should rest upon *fact;* and in the pursuit of this plan in studying the *phenomena and effects of fever*, modern pathologists have arrived at some of the most splendid results. They have abandoned, to a great extent, idle speculations as to the *proximate causes* and *exact nature* of fever; speculations that have always retarded, rather than advanced, the progress of medical science; and yet it is not perhaps affirming too much to say, as a starting point in our investigation, that all our Idiopathic or Essential forms of fever are the result of *the introduction of a poison of some kind into the system.*

The proof of this consists in the fact: 1st. That diseases analogous to these fevers have been induced by injecting putrid matter into the veins of animals: 2nd. These fevers are readily produced by the introduction of animal poisons into the blood, as in a case of small-pox, measles, &c.: 3rd. These poisons are known to operate through the medium of the air, by thus gaining access to the blood through the lungs: 4th. The non-contagious fevers, such as Intermittents and Remittents, are universally admitted to depend upon a poisoned or changed condition of the atmosphere: 5th. Actual observation establishes the fact that the blood *is* altered in all Essential or Idiopathic forms of fever.

That the febrile phenomena present in these fevers indicate a condition of the system *independent of inflammatory* action, I infer from the fact: 1st. That in the absence of complication during the progress of disease, there is no evidence of inflammation revealed by post mortem inspection: and 2nd. That the symptoms co-exist with a diminution of the fibrin of the blood, and diminished tolerance of the loss of blood. Evidently, therefore, the term *fever* may be used in two very different senses: in one signifying a collection of symptoms depending on local inflammation: and in the other a *condition* of the system entirely independent of such inflammation. In one the term indicates a *disease*, and in the other a *symptom*, just as head-ache, back-ache, furred tongue, and deranged secretions are symptoms. In the one kind, the local disease, if it be present, is consequent on the fever; in the other, fever is the result of the local disease. Hence the distinction between Essential and Symptomatic fevers. Let us take an example of each. A person in health is exposed to the contagion of an Enteric or Typhoid fever; he gradually becomes languid, stupid, has head-ache, back-ache, fever sets in, and during the course of the disease local symptoms manifest themselves, such as bronchitis, ulceration often of Peyer's glands, enlargement and softening of the spleen, &c. In the other case, a person, in equal health, from exposure to cold, or from local injury, gets an attack of pleurisy or inflammation of the lungs, and as a consequence, has Symptomatic fever from local inflammation.

Now it will be at once perceived that the relation which the fever sustains to the local disease in each of these cases is quite different. In the one the fever is *primary* and the local affection *secondary;* the fever will exist, therefore, although the local disease may be modified or removed. In the other the fever vanishes on the removal of the local disease.

A very able paper has been recently contributed on this subject by the distinguished Dr. Stokes. He divides all diseases into three classes. "In the first we have diseases which have an *anatomical character.* In the next we have a most important class of cases which have *no* anatomical character, and yet they are not fevers."

The first includes, of course, the *local phlegmasiæ*, and the symptomatic fever resulting therefrom. The second includes the *Neuroses*, such as mania, epilepsy, hysteria, lock-jaw, &c. It can not be shown that these depend upon any known or ascertained anatomical change of any portion of the system. That their seat is in the Nervous system is very evident, but what the condition of the nervous system is to which they owe their origin, the most minute and untiring observations of pathological anatomists have failed to point out. We are justified in affirming therefore, that the first class of diseases have no anatomical character. Now we come to the *third* class described by Dr. Stokes, which comprises *Fevers.*

The Idiopathic or Essential fevers are general, constitutional affections; they may be said to have no definite anatomical character. It may be asked, then, how do they differ from the *Neuroses?* Strictly speaking, they differ but little; they have many features in common; and often occupy an intermediate position between the strictly inflammatory and the purely neurotic affections.

Fevers differ, however, very much from the pure Neuroses in this, that fever seems to be a special condition of life, produced by the introduction of a *special poison* into the circulation, exists for a certain time, and only subsides on the elimination or neutralization of the poison.

They differ, also, from the pure Neuroses in their tendency to produce local anatomical changes *secondary to the fever.* The Neurotic affections do not tend to the developement of gastric hepatic, or bronchial disease, or to ulcers of the intestines, or cutaneous eruptions. These complications are not necessary, however, to the fever. We may have functional alterations occurring without any known organic change. Thus we have delirium in fever as a neurotic condition; pain as a neurotic condition; cough as a neurotic condition; and even abdominal tenderness may be present without enteritis or gastritis. We should remember, also, as a fundamental truth that lies at the basis of all correct views of fever, that a general morbid state is capable of destroying life *without any known anatomical change.*

The admittance of this doctrine into the field of pathology will enable us to obtain better and more definite ideas concerning the phenomena of fever; it will give us the power of striking at the root of disease; and, above all, it will banish from the Practice of Medicine much of that empiricism with which it has at all times been infested. It also offers the most rational explanation of the modification of diseased action growing out of prevailing *Epidemic influences.* It fixes the mind upon two controlling elements of disease; *first*, upon the depressing effects of a Zymotic poison operating upon the general constitution; *second*, upon the local inflammatory or functional complications that may be engrafted upon and influenced by a primary altered condition of the blood. It thus draws the line of demarcation between general and special pathology by keeping in view *constitutional conditions as modifying local action*, thus giving us broader views and clearer conceptions of the varied elements of disease as they act and react and insensibly shade into each other.

I have already intimated that the Essential fevers sustain an intermediate relation between the purely neurotic and the strictly inflammatory affections; and it may be well for our patients if, during the progress of the fever, it continues to maintain this intermediate position. But the tendency is to local and inflammatory complications. Thus we have, for instance, bilious, gastric, catarrhal and enteric fevers, by which we would not be understood to imply that there is nothing more than simply disease of the liver, stomach, respiratory system, or the mucous membrane of the bowels. These have been simply *superadded* to the general disease, and may constitute its most formidable feature. They are, in many cases, the cause of death, and may prove fatal either by the direct destructive effects of inflammation, or by preventing the efforts of nature towards a favorable termination.

Having premised these general reflections on the nature and pathology of the Essential fevers, I desire to call attention to the leading indications of *Treatment*, more especially of the Periodical and Autumnal fevers of the Mississippi Valley. And based upon the pathology which I have very briefly pointed out, what are the indications of cure? 1st. To counteract the injurious operation of the poisons: 2nd. To expel them from the system.

Unfortunately, however, we know of few remedies that will fulfil the first indication without injuriously affecting the blood itself. The second indication is the one most usually pursued, viz., to expel the offending matters from the system. This may be said to be Nature's mode of cure; and, in the absence of reliable knowledge as to the nature of the poison and its antidote, the physician can only aid nature in her work of elimination.

REMITTENTS.—I allude here to a type of miasmatic fever that has received numerous names, all of which are liable to objection. It is remittent—so are other fevers. It is also bilious—so is Yellow fever. It is essentially the same as Intermittent, produced by the same cause, having the same types, and amenable to the same treatment.

BLOOD LETTING.—An important question presents itself as to direct depletion by the lancet. Its propriety must, of course, be determined by the character of the symptoms and the constitution of the patient. What are the pathological differences between the Remittent and Intermittent varieties of fever? Evidently in the higher febrile excitement of the whole system; the greater tendency to visceral congestions and inflammations; and the much longer hot stage. We have a blending of a general and Essential fever with a local phlegmasia; and hence the want of complete intermission, as in intermittents. Remove the local lesion, and the antidote will take effect, as in ordinary intermittent. Blood-letting will often accomplish this object; but to be successful it should be resorted to at an early period, before inflammation in any organ is completely established. In young, robust, plethoric subjects, with hot skin, flushed face, severe pain in the head and back, and great gastric oppression, there ought to be no doubt as to the propriety of blood-letting. It will greatly facilitate the operation of other remedies, and often converts a Remittent into an Intermittent.

DIAPHORETICS.—If the skin remains hot, shall we resort to Dover's powder as a diaphoretic? Not while there is cold water. Have it applied according to Dr. Dixon's plan: "Set your patient in a convenient receptacle, and pour over his head and naked body from some elevation a stream of cold water until he is pale, or his pulse loses its fullness, or his skin becomes corrugated, or he shivers. Then have him dried and placed in bed, and copious perspiration usually follows." This is, of course, contra-indicated in the aged or feeble. But in young and vigorous subjects it is a most valuable therapeutic agent. The neutral mixture of the U. S. Dispensatory, variously combined with nauseants, may also be of service in promoting diaphoresis; and if the patient is restless, especially after the subduction of inflammatory excitement, a full Dover's powder should be administered at bed-time, and repeated if necessary to procure quietude and rest.

DIURETICS.—As a class of remedial agents in general febrile affections, diuretics are, I am well satisfied, too much neglected. There can be but little doubt that many of the constitutional disturbances characteristic of Idiopathic fevers are in a great measure due to defective excretions; and of the various excretory functions none equal in importance those of the Kidneys. These organs are in fact the great depurators of the system,—the exponents of the numerous chemical changes which are constantly taking place in the healthy and diseased animal fabric, and the principal channels through which foreign matters are expelled from the blood. Well selected diuretics, therefore, should hold a conspicuous place among our therapeutic agents in the cure of all forms of febrile disease.

PURGATION.—In withholding purgation in the treatment of the Autumnal fevers of the Mississippi Valley, I am aware that I come in conflict with high authority, and I would presume to question such authority, only from the conviction srongly impressed upon my mind, that active purgation is injurious in its tendencies and often fatal in its consequences. I doubt not but hundreds have fallen victims to erroneous views on this subject propagated by Hamilton in his work on Purgatives. Under the impression that "bilious disorder or hepatic derangements," as they are generally termed, play an important part in our Autumnal fevers, mercurial cathartics are used to a prodigal extent, and doubtless their effect has often been fatal. The pathological fact is lost sight of that the lesion of hepatic secretion is always a *secondary* condition; that it depends upon a certain *state* of the organ or of the system at large, and can be removed only by removing that state or condition. A clear conception of this fact would, I think, throw additional light on a class of diseases associated with derangement of the hepatic function, and banish from our nosology those numerous primary affections which are attributed to the liver.

What is the state or condition, therefore, of which the hepatic derangement is but an expression? First, congestion from intropulsion of blood, whether from the cold stage of an intermittent fever, or from protracted cold to the surface. The result of congestion from any cause, whether active or passive, is the lowering of the vital properties of the gland, and consequent suspension or perversion of secretion. Secondly, perverted secretion may result from a primary diseased condition of the blood itself. Or, lastly, we may adopt the explanation of Bichat, "that between a secreting organ and the surface upon which its excreting duct opens there is a sympathy by which a stimulus applied to the latter is communicated to the former." In either case the deranged secretion is secondary, to the *condition* producing it.

If these premises be correct, why do we administer purgatives for the relief of biliary derangement? I am aware that it is argued, theoretically, that the serous exhalation from the intestinal canal, caused by the action of a cathartic, unloads the vessels of the liver, and thereby restores its healthy circulation; and this argument might have weight were it not for the counteracting influence of irritation caused by the operation of the remedy. But this element of evil, I doubt not more than overbalances all the benefit derived from the depletion. In many instances the manifestations of biliary derangement are *produced* by irritation and phlegmasia of the mucous membrane. The illustrations of this are numerous. Errors of diet may give rise to it. A large quantity of indigestible food is taken at supper; next day the patient has vomiting, thirst, followed by jaundice. Or he may be exposed to heat, and, while suffering from lassitude and thirst, his body bathed in perspiration, he drinks large draughts of cold water. In a few hours he has nausea, thirst, fever, and awakes some morning and finds himself jaundiced. The same condition often follows drastic purgation. An increased irritation is communicated to the parenchyma of the liver, and whatever be the intensity of the phenomena attributed to the bile, *calmness is generally reestablished as soon as there is cessation of the local phlegmasia.* I regard this as an established fact in pathology of the highest importance.

In our ordinary Autumnal fevers, therefore, accompanied as they generally are with irritation of the stomach and bowels, I would abstain from the use of cathartics as calculated to aggravate the symptoms of biliary derangement and increase all the phenomena of the disease.

I would not, however, entirely exclude alvine evacuants in the treatment of these fevers. That their operation is often attended with benefit I would not for a moment deny. The acrid secretion may be a greater source of irritation, forward upon the mucous membrane and backward upon the gland secreting it, than would be the effect of a laxative to remove it. Or, if torpor or paralysis of the liver were present, associated with clay colored or white discharges, and unaccompanied by hyperæmia and tenderness of the gastro-duodenal mucous membrane, the *mercurial* cathartics would be admissible. But is it true, that, in the class of cases under consideration these are the manifestations? Is not, indeed, the very opposite condition generally present, such as local tenderness, irritability of the stomach, and dark discharges? Shall we, then, in this condition, administer cathartics? Many reasons forbid.

1st. As a general and valuable therapeutic principle we should never resort to medicinal agents when nature is doing her proper work.

2nd. Cathartics will increase the very difficulty which nature is endeavoring to overcome, by adding irritation and determination to the congestion already existing.

3d. There is no indication as a general proposition for their use, as evinced by the color and character of the discharges—dark discharges, characterising hyperæmia and effusion, positively contra-indicating their use.

SULPHATE OF QUININE.—All forms of our Autumnal fevers of the South and West—the Intermittent, Remittent and Congestive—are convertable one into the other; they possess one common property; there is but one feature, or element in either that is essential to their pathology, and that feature or element bows before the potent sway of the *sulphate of quinine.* The local lesions that take place during the progress of these fevers, are *secondary to this feature or element*, not primary and essential, and if this condition be not early arrested, fatal complications of vital organs may take place. Our first indication, therefore, should be to break up the paroxysms. We should not wait *for* remissions, but seek to *produce* them. In the malignant or congestive forms of intermittent fever, the timely administration of quinine in *large doses*, affords the only hope of the patient; it is the pivot on which the case is to turn for life or death.

I would not be understood, however, as regarding quinine the *only* means to be employed in the treatment of these fevers. If time admits, the intelligent practitioner will observe the usual preparatory treatment. He will also promptly counteract local determinations and complications. But the grand outstanding prominent idea to be cherished is the potency of the quinine to arrest the paroxysms.

In the inflammatory forms of remittent it may be necessary to premise by general blood-letting, or by the sedative effects of water applied by the dash, as suggested by Dr. Dixon, of Charleston, S. C.; or, what may even more successfully meet the same indication, cover the patient with a sheet, and have a sponge dipped in tepid water passed over every part of his body by the hour together, until the thermometer, both in the axilla and the mouth, has been brought down to 99 or 100 deg. The conditions of inflammatory excitement having been obviated, the antidote will be as prompt in its arrest of this as in the ordinary form of intermittent fever.

STRYCHNIA.—I regard this as a valuable adjuvant to the quinine in the malignant or congestive intermittents, especially if there be much difficulty in arousing the nervous system from its lethargy; and in the weak and broken down condition of the nervous system from an ordinary intermittent, it often proves to be a remedy of singular power.—*Western Lancet.*

